# To digitalize or not? Navigating and merging human- and technology perspectives in production planning and control

**DOI:** 10.1007/s00170-022-09874-x

**Published:** 2022-08-06

**Authors:** Kristina M. Eriksson, Linnéa Carlsson, Anna Karin Olsson

**Affiliations:** 1 Department of Engineering Science, University West, SE-461 86 Trollhättan, Sweden; 2 School of Business, Economics and IT, University West, SE-461 86 Trollhättan, Sweden

**Keywords:** Industrial digitalization, Smart manufacturing, Production planning control, Dynamic capabilities, Human-centric perspective

## Abstract

Contemporary manufacturing companies are navigating industrial digitalization anticipating increased production efficiency and competitiveness in a volatile environment. This study focuses on the implementation processes of digital tools for production planning and control (PPC), i.e., advanced planning and scheduling (APS) software, in relation to the application of analog planning with physical flow boards. Digital tools can support understanding the consequences of production changes and variations, hence facilitating adaptable and resilient manufacturing. However, technological changes can be daunting, and effective implementations require dynamic capabilities to remain competitive in elusive environments. The aim is to study the implementation processes of an APS software to understand the requirements of fruitfully moving from analog planning to next-generation digital tools for decision support in PPC. The paper presents an explorative case study, at a manufacturing company within the energy sector. The interview study took place over 9 months during 2020–2021, investigating current and retrospective aspects of the case across 2019–2021. The case study comprises 17 in-depth interviews with a range of company employees, e.g., logistics managers and functions responsible for digitalization development. The results highlight the challenges of implementing and especially trusting digital tools for PPC. To realize the value of digital tools for PPC, it is argued that it is imperative to simultaneously apply a human-centric perspective in decision making to ensure trustworthy, sustainable, and resilient human-data-technology nexus implementations towards smart manufacturing.

## Introduction

Manufacturing companies are attempting to navigate through industrial digitalization and adopting new digital tools anticipating improvement of their production efficiency and competitiveness in an evolving global economy. One area potentially benefitting from support of the application of digital technology within the era of Industry 4.0 (I4.0) is production planning and control (PPC) [1]. Nevertheless, manufacturing companies may struggle to strategically achieve long-term digital transformation due to difficulties understanding data generated from digital technologies and lack of competences [2]. Recently, Industry 5.0 (I5.0) has arisen, emphasizing the creativity and intelligence of humans in co-operation with intelligent machines to obtain user-favored manufacturing solutions [3, 4], thus, accentuating the human-centric perspective and the need to acknowledge employee involvement, striving towards trust, and new competence to achieve sustainable industrial digitalization when moving from manual to digital production planning and control.

PPC aims to deliver products on-time, in the correct amount, and in the right place to a suitable cost, and unbecoming PPC results in, e.g., high inventory, long waiting times, obsolescence products, and delays in deliveries [5]. PPC can be complex and stochastic when affected by disturbances [6]. Automated and digital tools for PPC, such as advanced planning and scheduling (APS) software, are designed to handle scheduling and rescheduling in dynamic shop floor environments to cope with breakdowns and material shortage [7]. Despite the long-time existence of digital PPC systems [8] and recent developments in I4.0, e.g., Internet of Things (IoT) and big data analysis, manually updated spreadsheets are still used to a large extent [9]. Over the past decades, lean production, a strategy of flow efficiency, has gained industrial interest [10]. More recently, some would argue that lean production cannot continue to be efficient without I4.0 technologies [11]. It is highlighted that I4.0 technologies, such as IoT and big data, are expected to be integrated with lean production, also stressing the human perspective as important for companies to succeed [12]. The evolution of PPC from Industry 3.0 through Industry 4.0 predicts the move towards adaptive, self-organized, big-data driven, collaborative, and digital twins-based scheduling [13]. Such planning requires a high digitalization level of manufacturing, e.g., real-time data collection and big data analysis. However, many manufacturers are still to move fully into the era of I4.0, as they have semi-automated production, older machinery, and a large amount of manual work [14, 15].

Further, certain production characteristics can make the use of I4.0 technologies complex, e.g., implementing fitting scheduling in high-mix low-volume manufacturing remains a challenge, as the properties of both parts and processes are dynamically changing [16]. Among criteria for real-time decision-support system for scheduling are IoT, inventory levels in real time, sensors, and actuators [16]. Big data analytics still have a low implementation level in manufacturing, emphasizing companies’ uncertainties for technical requirements and anticipated benefits of I4.0 [17]. Many traditional manufacturing companies have not reached a high digitalization level yet, turning to analog planning tools or manually handled spreadsheets. Thus, the matter of how manufacturing industry with low digitalization level can take advantage of novel technologies remains. In addition, it shall be realized that in the paradigm shift towards smart manufacturing, all are not able to move at the same pace, and the mindset of employees going through change needs to be considered [3, 15, 18]. Recent research put forth that manufacturing companies have disparate levels of digital maturity and other unique characteristics, e.g., different types and sizes of companies affecting the transformation and performance [19]. Thus, it is stressed that further interdisciplinary research is needed to study the phenomenon of industrial digitalization [19]. Therefore, it is relevant to understand how manufacturing companies can decide how to embrace digital technologies, or choose not to, yet stay competitive, while at the same time the aspects of the human-data-technology nexus are understood.

This study focuses on the implementation and application of an APS software in a manufacturing environment with predominantly manual work and a low level of digitalization and automation. The aim is to study the implementation processes of an APS software to understand the requirements of fruitfully moving from analog planning to next-generation digital tools for decision support in PPC. To understand the complexity of effective technology implementation, the study investigates the requirements through the lens of the Dynamic Capability Framework. The research question posed is: *How can the dynamic capability framework be applied to understand the transformative requirements for implementation of digital tools in production planning?*

Next, the framework for dynamic capabilities is outlined, followed by method and case description, data collection, analysis, results, discussion, and conclusion.

## Dynamic capabilities for digital transformation

The framework of dynamic capabilities (DC) was initially designed to explain how organizations achieve and sustain competitive advantages [20]. More specifically, the framework focuses on the actions taken by an organization to adjust current resources and to continuously adapt to and build advantage in elusive environments [20], here referring to industrial digitalization. DCs stem from a resource-based understanding of financial, technological, human, or supplier chain assets and the organizational capacity to modify resources [21]. Thus, the DC framework designed by [20] is adopted in this study and applied to highlight how an organization may fruitfully utilize its resources [22]. Moving from analog planning to next-generation digital tools for decision support in PPC implies changes to, e.g., company processes. Thus, it can be argued that understanding DCs is essential to implement novel technologies successfully [23].

DC comprises three core capabilities: sensing opportunities and threats, seizing opportunities, and transforming by organizing resources to companies’ envisioned utilization [24]. *Sensing* opportunities and threats involve activities such as scanning, creating, learning, and interpreting [24]. To carry out meaningful sensing activities, there is a need for the organizational routines to be related to identified underlying activities [23]. However, it may be significantly more challenging for established companies to predict and take advantage of novel technologies [25]. As envisioned, digital tools may challenge established standards, and changes in company processes may require structural changes to be captured [2]. *Seizing* opportunities relates to sensing capabilities as sensed opportunities need to be addressed through new products, processes, services, or a combination [24], hence, the capacity to capture the sensed value [23]. However, organizations frequently sense opportunities and threats but fail to seize value due to many reasons, such as lack of commitment, risk management, or financial concerns [24]. *Transforming* capabilities is the means of reconfigure organizational processes [26]. Transforming capabilities play an essential role in transforming existing resources to align with new strategies, building new resources, and supplementing current gaps in an organization’s resource base [26].

These comprehensive groups of core capabilities are widely used in the literature [20, 22–24, 26] and are applied in this study to analyze transformative requirements of implementing digital tools in production planning.

## Method

This section includes the case description, presentation of data collection, and an overview of informants, jointly with a description of the data analysis.

### Case description

One large Swedish manufacturing company in the energy sector was selected as the case, herein referred to as the Case Company (CC). CC produces and performs maintenance of cutting-edge, large-sized, heavy, high-quality components in a local and global chain of manufacturing units being part of a large global company. CC has a hierarchical organizational structure, operating at a centralized office level supported by the business functions. The industrial digitalization of the global company is scattered with advanced units as well as units in the early phases of digital transformation. CC has a low level of digitalization and automation with a high degree of manual work, e.g., welding operations. The factory layout is a job shop where similar processes are grouped. The large number of product variants share resources, are routed differently throughout the shop floor, and rework is often routed back to the shop floor, breaking up the production flow further. Those characteristics result in crossing flows and complex planning, which is difficult for employees to overview. In addition, job prioritization is affected by manually overriding production plans due to individuals’ experiences or pressing deadlines.

The case is framed around a production unit at CC, which is in its early phases of digitalization. The study focuses on implementing a digital PPC initiative, an APS software, which is set in relation to analog planning approaches. Characteristically for APS software is applying mathematics to calculate and analyze production schedules considering multiple constraints and performance measures. The intention is to demonstrate improved visibility for on-time delivery and inventory reduction.

### Data collection and analysis

This study was conducted as an explorative qualitative case study, focusing on the implementation processes to understand the transformative requirements of moving from analog planning to next-generation digital tools for PPC. A case study methodology was applied to capture the elusiveness of industrial digitalization by approaching it through semi-structured in-depth interviews with employees and observations in its real context [27, 28]. The term employee is herein applied in a general sense to describe all co-workers at the company. The qualitative approach allowed the informants to give voice to their understanding and interpretation [29] of the complexity when moving from analog to digital PPC.

To sample informants responsible for digitalization of production planning and control purposive sampling was applied. This choice of sampling was strategically made to capture informants’ perceptions and understanding of the phenomenon studied related to the research question [29]. Further, to identify informants, snowball sampling [29] was applied, meaning that one informant recommends the second who refers to the third and so on as a dynamic social process conveyed over time [30]. The phenomenon investigated is elusive and difficult to grasp and thus requires a sampling technique which allows to find hidden, hard-to-reach, and conflicting groups of informants [31].

The study took place over a period of 9 months (October 2020 – June 2021), investigating the case currently and in retrospective between the years 2019 and 2021. The case study comprises 17 in-depth interviews with technical, quality, production, and logistics functions, functions responsible for digitalization development, and corporate service functions such as HR, IT, and business administration, to get an encompassing understanding from different functions, see Table 1. In one of the interviews, two informants took part (13a, 13b); hence, a total of 18 informants are included. All interviews were digitally conducted due to restrictions of the Covid-19 pandemic and were recorded with informed consent.

**Table 1:** Overview of informants

Functions categorization	Number of interviews	Informant IDs	Duration (h)
Production planning and control	7	1, 2, 5, 9, 10, 12, 15	5.8
Technical management	2	3, 7	1.5
Quality management	2	4, 6	2.1
Digitalization development	2	8, 14	1.9
Business adm., controllers, HR	4	11, 13a, 13b, 16, 17	3.0
**Tot. 5 function categories**	**Tot. 17 interviews**	**Tot. 18 informants**	**Tot. 14.3 h**

**Fig. 1:**
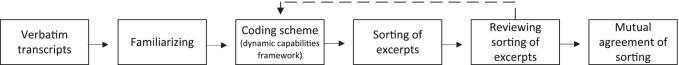
Flow chart of data analysis process

The qualitative approach yielded an analysis of different informants’ interpretation of production planning, either only planning or in relation to organization and digitalization. As illustrated in Fig. 1, the analysis followed a thematic analytical approach in which all collected data were verbatim transcribed. Then, the multidisciplinary group of authors familiarized themselves with the transcripts. Thereafter, the data was jointly analyzed in several rounds, by all authors, in an iterative approach following a thematic analysis [32].

Initially, 141 excerpts were selected in the collected data material of 14.3 h of recorded material. In the second sorting, excerpts directly related to the implementation of the APS software were picked out, resulting in 59 excerpts that were analyzed through the lens of the Dynamic Capabilities Framework core capabilities as: sensing opportunities and threats (31 excerpts), seizing opportunities (24 excerpts), and transforming (4 excerpts).

To reach a deeper insight into dynamic capabilities concerning the implementation of digital production planning tools, the authors were inspired by a narrative approach when collecting and analyzing the interview data and thus presenting the result as a narration revealing employees’ views and experiences [33]. The narrative approach to structure the results has been specifically chosen as this format gives emphasis to an interpretative and more descriptive understanding of the digitalization initiatives in the production planning and control, thus, unboxing the employees’ elucidations of the human-data-technology nexus implementations. After a mutual agreement of analysis, a total of 16 excerpts were selected as key to the narrative.

## Result and discussion

This section outlines the results narratively as a story told over time. The narration is followed by a discussion of what occurred during the narrative discussing how and why the story enrolled as it did.

### DC framework to understand the transformative requirements for implementation of a digital tools in production planning: a narrative

The results are narratively presented to explain and illustrate how CC moves back and forth through the phases of the DC framework as they strive towards improving PPC. Throughout the narrative, excerpts are included marked with the informants’ IDs and the phases of the DC framework that the excerpts refer to. The narrative covers the development of the PPC over a period of approximately 3 years, 2019 – 2021. As the data collection through the interviews began in 2020, informants reflect both on the current state and back in time. Also, organizational changes and restructuring of the management group took place during this period. CC has a traditional job shop layout, large product flora, a high rate of rework, and increasing order intake. CC has applied an Enterprise Resource Planning (ERP) system coupled with Excel spreadsheets over several years, and in parallel CC keeps those when introducing new specific planning approaches for PPC. Further, no approaches related to lean production, such as flow boards, 5S, or Value Stream Mapping, have previously been applied at CC.

**Fig. 2:**
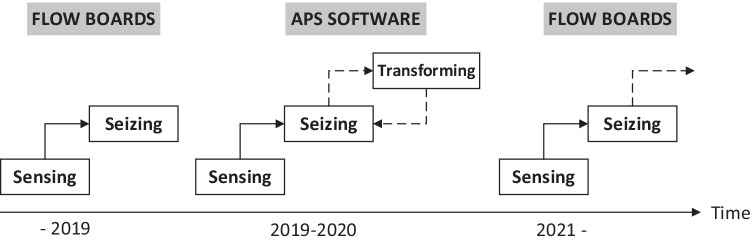
Schematic view of the DC phases that CC has gone through to address their PPC

Figure 2 outlines how CC moves stepwise and between the DC phases, showing the different initiatives of PPC over time. After an initial short trial with flow boards, CC implements an APS software and, in this case, partly reaches the phase of transforming but keeps returning to the seizing phase, which is shown as a loop between those phases, the dotted arrows in Fig. 2. Then, a decision is made to withdraw the APS software entirely. Thus, this digital tool is not implemented to its full use, and instead, CC decided to attempt anew to implement analog flow boards, see the right side of Fig. 2.

To begin the narrative, CC employees seemed aware of the move towards increased digitalization illustrated in the two following excerpts:

*“For me, digitalization is a paradigm shift… Today we are very document-based” *(8, Sensing).

*“Still [use] paper and pencil on the production floor”* (1, Sensing).

Further, the employees realized the planning complexity, and there seemed to be a consensus among employees at CC that PPC needed to be more efficient, illustrated in the following excerpts:

*“I think we are limited by our factory layout”* (4, Sensing).

*“We have about a thousand orders in progress, and we are four planners who will keep track of 250 orders each. No! It’s not possible! It’s inhuman”* (6, Sensing).

*“We have not acted on the (increase in) volumes we have seen”* (5, Sensing).

The above excerpts demonstrate the need for understanding improved PPC and CCs’ considerations to different approaches to improving PPC. Analog boards to visualize production flows and operations (flow boards) was the first approach to be tried, though this proved hard to implement, illustrated by the excerpts:

*“There was so much resistance to order, visualize and introduce the flow boards”* (2, Sensing).

*“The planning department said we cannot have the flow boards because they will mess up the APS software”* (2, Seizing).

Before the initiative with the flow boards developed into the DC framework’s transformation phase, this initiative was overridden by upper management’s decision to implement an APS software. The initiation of the APS software meant that CC retracted its steps to the sensing and seizing phases, as illustrated here:

*“In the beginning it [the APS] was not fully developed, so it also had a very large instability” *(4, Sensing).

*“The APS was updated once a day, but in one day quite a few changes in production can happen, it was not that flexible”* (15, Seizing).

Going into the phase of seizing, CC are struggling to adjust and follow the plan by the APS software; the excerpts below show movement between the phases of seizing and transforming:

*“One challenge is that everyone should believe in the plan, so that you actually do what is planned. The next challenge is that those who are in a hurry and do not want to follow [the plan] stand last in the queue”* (6, Seizing).

*“The APS software was very, very good, I think. The downside to it was that it was not updated live.”* (4, Transforming).

*“It is always inconvenient when a system says that it actually looks this bad. And that acceptance is a company not always is ready to take” *(11, Transforming).

In relation to this, a point recurs in the interviews of the difficulty of finding time to work with improvements—a point that increases the risk of getting stuck in the sensing phase, illustrated by:

*“We do not have time to work with what might be very value-creating for the customers”* (8, Sensing).

The implementation of the APS software proved difficult due to the disinclination to trust the APS software, and thus orders were prioritized outside the APS software plan. Though some believe the APS software could have worked if there was a consensus to follow it, but as it turned out the planning made by this software was constantly overridden. This lack of trust may indicate why CC gets stuck in between the DC phases seizing and transforming. Eventually, this led to the removal of the APS software and thus transported CC’s attempts of improving PPC back to the sensing phase, and instead a second try with analog flow boards was embarked upon. When we leave the narrative, CC has reached the seizing phase with analog flow boards as PPC method, illustrated by the following excerpts:

*“We have gone back in development to moving operations on a flow board manually. It became easier and clearer…because it gave a sense of safety”* (7, Seizing).

*“It’s about getting employees to understand the big picture. Where are we going? And understand your role in the production flow… you must visualize the production flow to understand your role in this”* (15, Seizing).

*“We do not master our processes, but if we learn our processes, we will constantly get better”* (14, Sensing).

When we leave the narrative CC is moving towards the transforming phase with the flow boards approach.

## Discussion

Throughout the interview study, there seemed to be a consensus among employees that CC needs to improve their production planning and control, especially as it is highlighted that production variations are significant, and the order intake is increasing. However, there are different ideas of how and what approaches to improve PPC that should be chosen. CC seems to lack a coherent view of how to approach the planning and what strategies to work towards as they move between analog and digital approaches. To sense activities towards improving PPC, there is a need for embedded organizational routines related to underlying activities [23, 24]. The employees emphasize that aspects such as crossing production flows and disparate products variants make planning difficult, and they request a better understanding of how to manage such production characteristics.

Consequently, the scattered understanding of the production traits may make it more challenging to succeed with the implementation of novel approaches and digital tools if the holistic aspects of the production characteristics are not fully grasped. In other words, the routines related to underlying activities are not clearly defined. For traditional manufacturing companies, sensing capabilities to predict, take advantage of, and implement new approaches and novel digital tools could be even more challenging due to organizational historicity [25], as shown in CC. This underlines the importance of communication for a shared understanding and joint identified strategies of the planning needs before embarking on new approaches and novel digital tools. If one does not grasp the production traits, one cannot seize to implement and apply a new planning system. Further, the consequences of overriding the plan by manual re-prioritization need to be realized, as such undertakings may result in production backlog, overwork, and rework.

After applying the digital planning solution of an APS software, CC retraces its steps and begins anew with an analog approach of physical flow boards in production. Reconfiguring means is argued to be imperative for transforming existing resources and activities [19, 23]. Indeed, CC retracing the steps may be a reaction to recognizing the importance of understanding the production characteristics before embracing digitalization. However, manufacturing companies fail to seize the value for many reasons, such as lack of commitment, aversion to risk, or financial reasons [21, 24]. If the manufacturing setting has a low automation level and mainly manual work, is it necessary to begin with an analog planning approach to move forward? It can be argued that this is not the case; somewhat digital technologies can make lean production more effective and reduce its limitations, such as poor customization [12], and improve flexibility and productivity [13]. In the case described here, CC returned to an analog lean approach. An advantage may be that it enhances employee involvement and thus supports an improved understanding of the production. If this is the case, then the nexus between humans and technology is missing, arguing that understanding the production characteristics should be facilitated from a human-centric perspective [2]. This since traditional manufacturing companies may find it much more challenging to adapt to standardized technological solutions [4]. CC failed to implement analog flow boards the first time around, running into much resistance and the incentive did not seem strategically anchored. Thus, the company culture of resisting change, scattered trust in the PPC system, and overriding planning decisions have impacted analog and digital approaches, e.g., manifesting challenging historicity within CC [25]. Rather than working scattered across the production job shop and risking sub-optimization, it is vital with coherent and holistic strategies, thus, mutually working towards continuously keeping fruitful communication between employees to build and sustain an adaptive community [15].

**Fig. 3:**
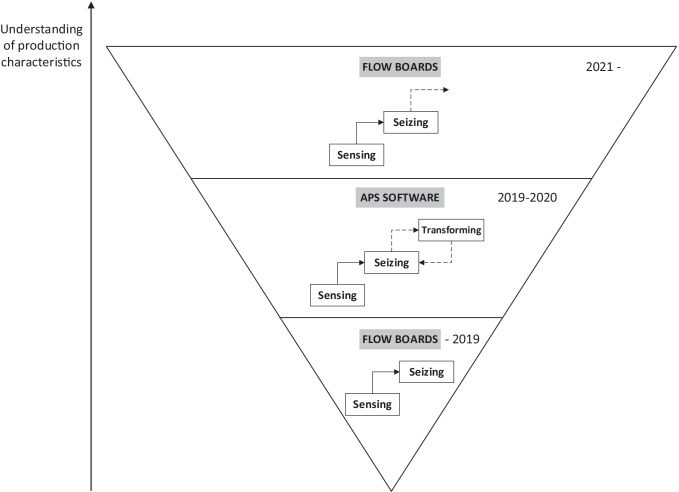
Overview and analysis of the DC phases that CC has gone through to address their PPC illustrating the increased understanding of CC’s production characteristics

In this case, reversing to analog planning can indicate a higher level of comprehension of their production characteristics. CC may have realized the need to find solid ground before reconfiguring their search for and navigation through the winding roads of industrial digitalization. To illustrate this, Fig. 3 displays the DC phases from bottom to top in a triangle shape with an arrow in the Y axis pointing upward to visualize and highlight the increasing understanding of the production characteristics. The triangle in Fig. 3 is divided in three areas where the DC phases that CC has gone through to address their PPC are shown from bottom to top and indicating the time frame in years in each area and thus emphasizing that the understanding is higher in relation to historicity. The three areas of the triangle are increasing in size in the upward direction, symbolizing the increased understanding of the production characteristics throughout the different attempts of addressing PPC and moving through the DC phases. To emphasize the upward pointing arrow shows the higher and broader, i.e., more holistic, understanding of the PPC needs for CC’s production characteristics, hence, accentuating a cumulative knowledge building of identifying production characteristics in relation to introduction of new techniques for production planning and control in the organization.

To summarize, this study has outlined a case of finding suitable solutions to PPC, studying the implementation of an APS software in relation to an analog approach with flow boards. The data collection from the interview study was analyzed through the Dynamic Capability Framework indicating the phases of sensing, seizing, and transforming to understand the processes of change in the era of digital transformation. As a response to the technocratic understanding of industrial digitalization, a more human-centric perspective, Industry 5.0 (I5.0), has arisen [4], thus, reasoning that industrial digitalization requires human-centricity and incremental steps of change when implementing new incentives [15]. This is illustrated by the results herein; when aiming towards successful, sustainable, and resilient production planning and control it is essential to apply clear and common strategies in which the human-centric perspectives are in focus. The use of digital tools for PPC shall not be discarded, especially as the study demonstrates implications of what is required to succeed in the next iteration of industrial digitalization.

## Conclusion

The DC framework has been applied to study transformative requirements for implementing digital tools for PPC. The study contributes a deeper insight into how traditional manufacturing companies navigate industrial digitalization, struggling to merge human and technology perspectives while moving from manual to digital production planning and control. Enriching for the study has been the multidisciplinary research group setting of authors with expertise and perspectives from production systems and logistics, information systems, and organizational sciences, contributing to a multifaceted understanding of the studied phenomenon.

This study concludes that, to link human and human, and human and technology in manufacturing corresponding to the emerging Industry 5.0, it is vital that employees understand the production and its characteristics before embracing new technologies. If the production characteristics are unclear to company employees, the company can neither sense or seize how to use new technology, nor be capable of trustworthy decision-making to transform production planning and control in a sustainable and resilient manner. Furthermore, employees have limited understanding of the overall consequences of not following the plan, e.g., the effect of overwork, delays, quality issues, rework, and manually re-prioritizing orders. Thus, there seems to be a prerequisite ability to grasp and comprehend the holistic view and understanding of the production. Potentially, this can be overcome by avoiding employees working in scattered clusters. Arguable, the importance of communication between employees on all levels to jointly understand the production characteristic and the company’s needs is essential. Moreover, it is recognized that industrial digitalization needs incremental steps when implementing digital production planning and control. Simply digitalizing industrial processes does not necessarily generate a system that will automatically give a prosperous production. This study clearly shows that employees need to understand the holistic production system before digitalization. This aspect is essential to grasp, by all employees, as part of the development towards increased collaboration of human intelligence and intelligent machines that is the focus of Industry 5.0. The added aspect of human-centricity is especially vital in traditional, low-level digitalized manufacturing, thus, concluding that the nexus between human and technology cannot be dismissed when deciding whether to digitalize or not.

There may be limitations because of the single case study and the sampling technique applied; thus, it is encouraged in future studies to cover more functions and/or multiple cases. The intent of this study was to identify employees and functions relevant for the posed research question and to put forth the in-depth understanding through a narrative approach highlighting the human-technology nexus contributing to the emerging I5.0 research. Future work in this field is encouraged to take a human-centric perspective when studying and understanding how the manufacturing industry and, production planning and control, can become sustainable and resilient.
